# Test–retest and interrater reliability of experimental within-subject variability of pain reports as assessed by the focused analgesia selection test

**DOI:** 10.1097/PR9.0000000000001175

**Published:** 2024-08-16

**Authors:** Mariana Agostinho, Adi Shani, Rita Canaipa, Roi Treister

**Affiliations:** a The Cheryl Spencer Department of Nursing, Faculty of Social Welfare and Health Sciences, University of Haifa, Haifa, Israel; b CIIS, Centre for Interdisciplinary Health Research, Faculty of Health Sciences and Nursing, Universidade Católica Portuguesa, Lisbon, Portugal; c Department of Orthopedics B and Spine Surgery, Galilee Medical Centre, Nahariya, Israel; d Oncologic Day Care Unit, Galilee Medical Centre, Nahariya, Israel

**Keywords:** Baseline pain fluctuations, Clinical trials, Variability, Placebo, Quantitative sensory testing

## Abstract

**Introduction:**

Within-subject variability (WSV) of pain intensity reports has been shown to predict the placebo response. The focused analgesia selection test (FAST), which allows to experimentally assess WSV of pain reports, has been used as a screening tool to identify participants who are likely to have a strong placebo response in drug-development clinical trials. Yet, the reliability of FAST has not been reported.

**Objectives:**

To assess test–retest and interrater reliability of the FAST outcomes. To mimic pharma-sponsored clinical trials, we enlisted inexperienced assessors who underwent limited training.

**Methods:**

Healthy volunteers performed the FAST twice within a week and were randomly assigned to either the test–retest group or the interrater group. *T*-tests, partial Pearson correlations, intraclass correlations (ICC), and Bland–Altman plots were generated to assess FAST outcomes' reliability.

**Results:**

Sixty-three participants completed the study and were assigned to the test–retest (N = 33) or interrater (N = 30) arms. No statistically significant differences in the FAST outcomes were detected between the 2 sessions, except for the FAST covariance (FAST *CoV*) in the interrater assessment (*P* = 0.009). Test–retest reliabilities of the FAST-main outcomes were r = 0.461, ICC = 0.385 for the FAST *R*^2^ and r = 0.605, ICC = 0.539 for the FAST ICC and in the interrater cohort, they were FAST *R*^2^: r = 0.321, ICC = 0.337 and FAST ICC: r = 0.355, ICC = 0.330.

**Conclusion:**

Using inexperienced assessors, the FAST outcomes test–retest ranged from moderate to strong, whereas the interrater reliability ranged from weak to poor. These results highlight the importance of adequately training study staff members before using this tool in multicentre clinical trials.

## Abstract 2

Within-subject variability of pain reports, as captured by the FAST, demonstrates moderate reliability, comparable to the reliability of other dynamic quantitative sensory testing measures.

## 1. Introduction

Pain is a dynamic, subjective experience that often fluctuates from moment to moment.^39^ In recent years, there has been a growing interest in the intraindividual fluctuations of pain reports.

To the best of our knowledge, Harris and colleagues were the first to identify the clinical relevance of the Within-subject variability (WSV) of clinical pain through the analysis of clinical pain diaries in patients with fibromyalgia. In this retrospective analysis, the WSV of pain intensity reports predicted the placebo response: the greater the WSV, the stronger the placebo response.^22^ Subsequent studies highlighted similar results across various clinical populations,^6,12,34^ including a meta-analysis of 12 clinical trials (N = 2740) of patients suffering from postherpetic neuralgia and painful diabetic peripheral neuropathy.^17^

Another experimental approach to study the WSV of pain intensity reports is through the focused analgesia selection test (FAST).^40^ The FAST is a psychophysical test based on quantitative sensory testing (QST) methodology,^21^ allows researchers to quantify the WSV of pain intensity reports in response to noxious stimuli of various intensities. Significant individual differences in the variability of pain reports, measured through the FAST outcomes, have been observed in healthy individuals and chronic pain patients^1,10,11,40^ Clinical trials held by our research team and collaborators have revealed the clinical significance of the FAST outcomes, linking them to changes in clinical pain after exercise (eg, climbing stairs),^40^ the variability of clinical pain reports recorded in pain diaries, and the placebo response.^41^

Until recently, the explanation for the associations between the WSV of pain intensity reports, either experimental or clinical, with the placebo response remained elusive. Recently, we provided a possible explanation, which is based on the Bayesian brain hypothesis.^25^ It suggests that pain perception is a predictive process^9,15,25^ in which the brain could be viewed as a predictive machine, continuously evaluating and weighing prior expectations (“priors,” in Bayesian terminology) against incoming sensory information (the “likelihood”). The perceived pain will be more strongly influenced by the factor that carries higher certainty. For this reason, subjects with large WSV demonstrate a strong placebo response because their certainty of their bodily signals is low. Hence, their subjective perception will be largely influenced by top-down processes (the priors). By contrast, those who demonstrate small WSV are more certain of their bodily signals than of their priors and will therefore be less affected by the placebo. To conclude, our hypothesis proposes that the fluctuations in pain reports (whether observed in clinical or experimental settings) may serve as a surrogate measure of the certainty an individual assigns to the ascending sensory information in reliably providing information on pain states (ie, the likelihood) and may reflect individuals' confidence in interpreting their own bodily sensations.

In an attempt to minimize the placebo response in clinical trials,^19^ pharma-sponsored trials have adopted the FAST as an exclusion criterion to identify and exclude subjects who are expected to have a strong response to placebo. Two studies used the FAST to assess the analgesic potential of 2 different transient receptor potential vanilloid type 1 (TRPV1) receptor antagonists.^4,28^ Despite the small cohorts including 32^28^ and 54^4^ patients, both of these phase II studies resulted with significant results. Although these studies were not designed to assess the added value of using the FAST as a screening tool in terms of assay sensitivity, a small cohort nevertheless yielded positive results in both these studies, suggesting that using the FAST might have contributed to the positive results of these trials.^4,28^

The reliability of the FAST has never been reported, despite its adoption in multicentre, pharma-sponsored, clinical trials. Because the staff members in the participating clinical sites have had no previous experience in applying psychophysical tests, we measured the test–retest and interrater reliability of the FAST conducted by inexperienced assessors. Our aim was to mimic a real-world application of the FAST to assess its reliability in ecological manner.

## 2. Methods

### 2.1. Participants

Healthy volunteers from local Haifa universities were recruited through various channels, including flyers, social media, and the university pool of participants. Inclusion criteria were adults (older than 18 years) who understand spoken and written Hebrew or Arabic; had no chronic pain condition, psychiatric, cognitive, or neurological disorders; had no history of alcohol or drug abuse/dependence; had no regular intake of medications, apart from oral contraceptives; and had no consumption of analgesics 48 hours before laboratory visits. Written informed consent was obtained at the first study visit. A set of sample size calculations based on correlations and repeated measures models was conducted using G*Power 3.1.9.7 software,^18^ focusing on a significance level of 5%, a power of 0.8, and an effect size of 0.4 (correlation coefficient |p|). These calculations indicated that the sample size needed to meet the desired statistical power is N = 34 in each analysis (test–retest and interrater).

### 2.2. Focused analgesia selection test

In the FAST procedure, a total of 49 thermal stimuli of 7 varying intensities (44°C, 45°C, 46°C, 47°C, 48°C, 49°C, and 50°C) are applied to the participant's nondominant forearm ventral surface, using a Medoc TSA-2 thermode (30 mm^2^ × 30 mm^2^, Medoc TSA-2001 device, Ramat Ishai, Israel). Each stimulus intensity is applied 7 times in random order, with a 15-second interval between the stimuli. The thermode is adjusted to adjacent site of the ventral forearm every 10 stimuli to minimize habituation and/or sensitization effects. Stimuli characteristics involve a temperature increase from baseline (32°C) to 1 of the 7 defined temperatures for 3 seconds, followed by a return to baseline, lasting a total of 8 seconds (ramp up and down, with slightly different ramping up and down rates to adjust a fixed stimulus duration of 8 seconds). The participants rate their perceived pain intensity on a numerical rating scale (NRS) from 0 to 100 (where 0 = “no pain” and 100 = “the worst pain imaginable”) in response to each stimulus. The procedure begins with familiarizing participants with noxious heat stimuli (43°C, 45°C, 47°C) followed by the FAST procedure (total duration of 25 minutes).

The FAST allows the computation of 3 outcomes using distinct statistical models: (1) The Pearson coefficient of determination (R^2^) was based on a power regression of the psychophysical function of pain intensity ratings vs stimulation temperature, indicating the proportion of variability. A higher FAST *R*^2^ reflects lower WSV in pain ratings, and vice versa (a lower *R*^*2*^ reflects higher variability). (2) The FAST intraclass correlation coefficient (FAST ICC) is calculated using a 2-way mixed-effects model of absolute agreement. It measures absolute agreement for the 7 administrations of each stimulus intensity. A higher ICC denotes lower WSV. (3) The coefficient of variation (*CoV*) is the ratio of the standard deviation to the mean, calculated for each intensity and then averaged across intensities. Higher *CoV* values indicate larger variability in pain reporting.

### 2.3. Study design

The study protocol has been approved by the University of Haifa ethical committee (approval number 186/22). To achieve the study objectives of assessing the test–retest and interrater reliability of the FAST, a within-subject repeated-measures design was employed. Two students without any previous experience in data collection or in QST were recruited from the Psychology Department of the university of Haifa. Before data collection and after completing a formal Good Clinical Practice course, the 2 students underwent a short training program focusing on ethical guidelines, equipment usage, and procedural consistency to ensure uniformity in instructions and application procedures. We intentionally chose to recruit inexperienced assessors, to simulate how multicentre, pharma-sponsored, analgesic, clinical trials are conducted in the real world.

Afterward, the recruited participants attended 2 sessions 1 to 2 weeks apart. Half of the participants were randomly assigned to the test–retest group, with both sessions being conducted by the same assessor. The other half were assigned to the interrater group, in which each session is conducted by a different assessor. During each session, the participants underwent the FAST procedure. At the end of the second session, all the participants received compensation for their participation.

### 2.4. Statistical analysis

The data analysis was conducted using SPSS Statistical Package for the Social Sciences software (IBM SPSS Statistics, version 25.0) and visualization with R studio (version 1.1.463; R version 3.5.3). Descriptive statistics were used to assess sociodemographic and main variable distributions. Normality was assessed using the Shapiro–Wilk test, followed by kurtosis and skewness inspection. Although raw pain reports (ie, pain sensitivity) distributed non-normally, the FAST outcomes distributed normally. For this reason, we used nonparametric tests to assess pain sensitivity–related analyses, and parametric tests to assess the reliability of the FAST main outcomes.

All analyses were employed separately for test–retest and interrater cohorts. Friedman tests were used to assess differences in median pain intensity reports across different stimulus intensities. Subsequently, post hoc analyses were performed using the Wilcoxon-signed rank test for paired samples to compare differences between median pain intensity reports across each pair of stimulus intensities. These analyses were applied to pain sensitivity outcomes of session 1 and session 2 separately, to assess if participants distinguished between intensity of stimulation in each session.

Both for test–retest and interrater cohorts, we compared disparities in median pain intensity reports across sessions for each stimulus intensity. To achieve this, we used the Wilcoxon signed-rank test with Bonferroni correction for multiple comparisons. With the Bonferroni correction considered, the threshold for significant results in these analyses was set at 0.007 (alpha = 0.05 divided by 7).

To analyse the FAST outcomes, parametric analyses were used. Paired *t*-tests and Cohen d effect size were employed to assess between session mean differences of the FAST outcomes. Partial Pearson correlations with time between sessions as a covariate were used to assess test–retest and interrater reliability of the FAST outcomes. Reliability boundaries were set at <0.20 (very weak), 0.20 to 0.39 (weak), 0.40 to 0.59 (moderate), 0.60 to 0.79 (strong), and >0.80 (very strong).^16^ Intraclass correlation coefficients, measured as 2-way random effects, absolute agreement, single measurement, with 95% confidence intervals (CIs) were also calculated. The ICC results indicated the following levels of reliability: <0.4 (poor), 0.4 to 0.59 (fair), 0.6 to 0.75 (good), and >0.75 (excellent).^21^ Statistical significance was defined as *P* < 0.05. Finally, Bland–Altman plots illustrated limits of agreement (LoA), displaying the relationship between the mean of the 2 measurements against the difference between the measurements.^7^ The mean difference represents the bias, and the plots were interpreted as the majority of the points required to be around the bias line, within the 95% CI LoA. Lower bias and LoA signify closer values between the measurements, indicating better reliability. An even distribution across the Bland–Altman plots indicated no evidence of bias.

## 3. Results

### 3.1. Participants' characteristics

A total of 75 participants initially volunteered to take part in the study, and 63 participants were included in the final analysis. Four of the initial 75 participants withdrew due to pain sensitivity, 5 were lost to follow-up after the first session, and 3 participants were excluded from the final analysis because they took analgesics within 48 hours preceding the laboratory visits.

Of the 63 participants, 39 were female. The mean (SD) age of all participants was 25.9 (6.8) years, ranging from 18 to 59 years. More than half of the participants (55.6%) had more than 15 years of education. The second session occurred an average of 8.40 (3.71) days after the first session. Among the 63 participants, 33 underwent the FAST with the same assessors (test–retest) in the first and second sessions, and 30 underwent FAST with different assessor (interrater) in each session.

### 3.2. Pain sensitivity

#### 3.2.1. Differences in pain sensitivity for test–retest and interrater reliability

**Figure 1.**
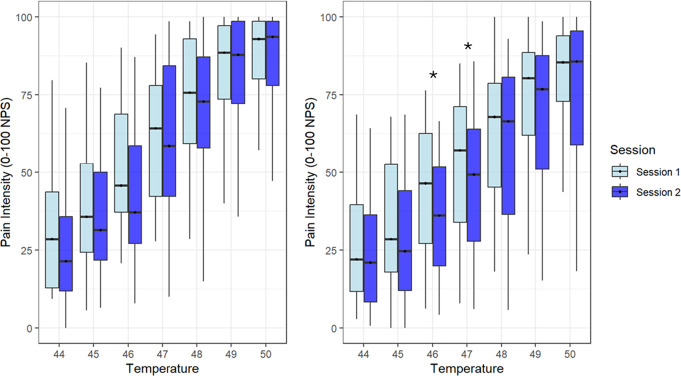
Median pain intensity of the FAST stimuli across sessions presented for each cohort (left side the test–retest reliability cohort; right side the interrater cohort). The boxplot represents the interquartile range (IQR), which spans from the lower to the upper quartile. The central line within the box denotes the median. *P < 0.05. FAST, focused analgesia selection test.

In the test–retest reliability cohort (Fig. 1, left panel), median pain scores (interquartile range [IQR]) in the first session ranged from 28.57 (30.71) in response to the lowest stimulus (44°C) to 92.86 (18.57) for the highest stimulus intensity (50°C). In the second session, median pain scores ranged from 21.43 (23.86) to 93.57 (20.71) for the lowest stimulus (44°C) and highest stimulus intensity (50°C), respectively. Similarly, in the interrater cohort (Fig. 1, right panel), median pain scores in the first session ranged from 22.07 (27.68) to 85.36 (21.07) for the lowest (44°C) and highest stimulus intensity (50°C), respectively. In the second session, median pain scores ranged from 21.07 (27.68) to 85.71 (36.43) for the lowest stimulus (44°C) and highest stimulus intensity (50°C), respectively.

To assess differences in pain intensity reports between stimulus intensities, Friedman's test for the test–retest (session 1: χ^2^ (6) = 184.629, *P* < 0.001; session 2: χ^2^ (6) = 184.92, *P* < 0.001) and interrater reliability cohorts (session 1: χ^2^ (6) = 168.00, *P* < 0.001; session 2: χ^2^ (6) = 170.33, *P* < 0.001) revealed significant differences in median pain intensity scores across stimulation intensities. Post hoc analysis using Wilcoxon signed-rank tests showed significant differences between all stimuli intensities (*P* < 0.001 for all comparisons) in each session for both cohorts. These findings underscore that at the group level, participants were able to distinguish between stimuli intensities.

To assess differences in pain intensity reports between sessions, for both the test–retest and the interrater cohorts, we used the Wilcoxon signed-rank test with Bonferroni correction. In the test–retest cohorts, no significant differences were observed. In the interrater reliability cohort, significant differences in pain intensity reports were found in the 46°C (Z = −3.731, *P* < 0.001) and the 47°C (Z = −2.877, *P* = 0.004) stimuli. These results suggest that other than these 2 exceptions, pain sensitivity was generally stable between both sessions.

### 3.3. Comparison of within-subject variability of pain reports between both sessions

Paired *t* test was used to assess between sessions differences in the FAST main outcomes (Table 1). No significant differences were observed in the FAST *R*^2^ in either the test–retest cohort (t(32) = −0.732 *P* = 0.470 CI [−0.072, 0.034], d = 0.150) or the interrater cohort (t(29) = −1.472 *P* = 0.152 CI [−0.111, 0.018], d = 0.172). Similarly, no significant differences were observed in the FAST ICC in the test–retest cohort (t(32) = −1.745 *P* = 0.091 CI [−0.072, 0.006], d = 0.110) and the interrater cohort (t(29) = −1.388 *P* = 0.176, CI [−0.110, 0.021], d = 0.175). Significant differences were found only in the *CoV*, which demonstrated significant differences in the interrater assessment (t(29) = −2.811 *P* = 0.009, CI [−0.156, −0.025] d = 0.176) but not in the test–retest cohort.

**Table 1:** Descriptive and differences between sessions of the focused analgesia selection test outcome for test–retest and interrater reliability.

Test–retest									
N = 33	Session 1			Session 2			Difference (T1 − T2)		
	Mean (SD)	Median	Range	Mean (SD)	Median	Range	*t*	*P*	Cohen d
*R* ^2^	0.53 (0.13)	0.54	0.28–0.82	0.54 (0.14)	0.52	0.25–0.80	−0.732	0.470	0.150
ICC	0.66 (0.12)	0.70	0.39–0.89	0.70 (0.12)	0.71	0.42–0.88	−1.745	0.091	0.110
CoV	0.38 (0.21)	0.34	0.06–0.99	0.36 (0.16)	0.37	0.06–0.77	0.264	0.794	0.274

Interrater									
N = 30	Session 1			Session 2			Difference (T1 − T2)		
	Mean (SD)	Median	Range	Mean (SD)	Median	Range	*t*	*P*	Cohen d
*R* ^2^	0.53 (0.16)	0.58	0.15–0.78	0.58 (0.13)	0.59	0.33–0.82	−1.472	0.152	0.172
ICC	0.64 (0.17)	0.69	0.17–0.85	0.69 (0.13)	0.67	0.42–0.94	−1.388	0.176	0.175
CoV	0.34 (0.15)	0.31	0.11–0.66	0.43 (0.24)	0.39	0.08–1.07	−2.811	0.009*	0.176

CoV, covariance; ICC, intraclass correlation coefficient.

### 3.4. Assessment of test–retest and interrater reliability of the focused analgesia selection test outcomes

Table 2 summarises the results for test–retest (top) and interrater (bottom) reliability measures calculated for each outcome of the FAST. For the test–retest cohort, the FAST *R*^2^ and the FAST ICC showed statistical significance for all reliability measures. The FAST *R*^2^ partial Pearson correlation was *r* = 0.461 (*P* = 0.008, CI [0.154, 0.679]) and showed a reliability of ICC = 0.385 (*P* = 0.031, CI [0.052, 0.641]). The FAST ICC outcome showed a correlation of *r* = 0.605, *P* < 0.001, CI [0.387, 0.765] and a reliability of ICC = 0.539, *P* < 0.001, CI [0.253, 0.740]. On the other hand, the FAST *CoV* showed a correlation of *r* = −0.083, *P* = 0.650, CI [−0.428, 0.467] and a reliability of ICC = −0.032, *P* = 0.570 CI [−0.381, 0.317], both not significant.

**Table 2:** Test–retest and interrater reliability measures.

Test–retest							
FAST	Correlation			ICC			LoAs
	*R*	*P*	95% CI	ICC	*P*	95% CI	Lower LoA − upper LoA
*R* ^2^	0.461	0.008	0.154, 0.679	0.385	0.013	0.052, 0.641	−0.313, 0.275
ICC	0.605	<0.001	0.387, 0.765	0.539	<0.001	0.253, 0.740	−0.250, 0.182
CoV	−0.083	0.650	−0.428, 0.467	−0.032	0.570	−0.381, 0.317	−0.524, 0.550

Interrater							
FAST	Correlation			ICC			LoAs
	*R*	*P*	95% CI	ICC	*P*	95% CI	Lower LoA − upper LoA
*R* ^2^	0.321	0.089	−0.067, 0.652	0.337	0.029	−0.007, 0.614	−0.384, 0.291
ICC	0.355	0.059	−0.045, 0.677	0.330	0.032	−0.016, 0.609	−0.388, 0.299
CoV	0.688	<0.001	0.510, 0.827	0.566	<0.001	0.240, 0.772	−0.436, 0.255

FAST, focused analgesia selection test; ICC, intraclass correlation coefficient.

For the interrater cohort, the FAST *R*^2^ exhibited a correlation of *r* = 0.321, *P* = 0.089, CI [−0.067, 0.652], with a reliability of ICC = 0.337, *P* = 0.029, CI [−0.007–0.614], whereas the FAST ICC demonstrated a correlation of *r* = 0.355, *P* = 0.059, CI [−0.045, 0.677] and reliability of ICC = 0.330, *P* = 0.032, CI [−0.016, 0.609]. The FAST *CoV* outcome showed statistically significant correlation and reliability between the 2 sessions on the interrater assessment with *r* = 0.688 (*P* < 0.001, CI [0.510, 0.827]), and a reliability of ICC = 0.566 (*P* < 0.001 CI [0.240, 0.772]).

### 3.5. Bland–Altman plots and limits of agreement for test–retest and interrater reliability

**Figure 2.**
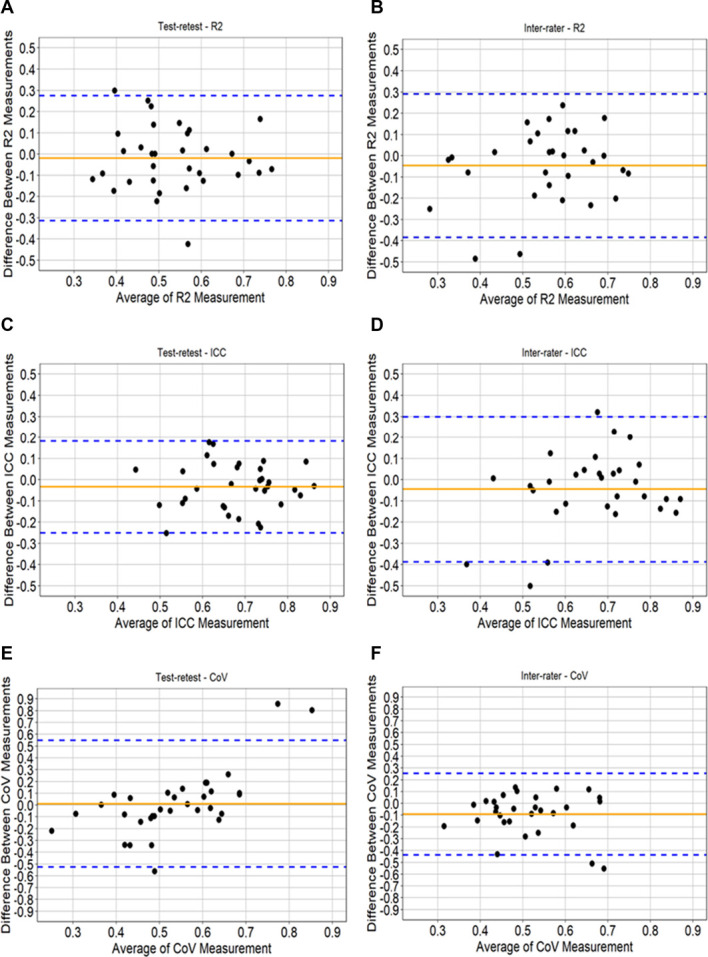
A–F. Bland–Altman plots of the test–retest and interrater reliability for *R*^*2*^ (A and B), ICC (C and D) and *CoV* (E and F). CoV, covariance; ICC, intraclass correlation coefficient.

Figure 2A–F depict the Bland–Altman plots and limits of agreement (LoA) (also in Table 2) for FAST *R*^2^, FAST ICC, and FAST *CoV* for each participant, assessing both test–retest and interrater reliability.

The Bland–Altman plots for test–retest reliability show similar small limits of agreement for both FAST *R*^2^ (−0.313, 0.275) and FAST ICC (−0.250, 0.182), with the average mean difference close to 0 and no systematic bias evident. For FAST *CoV*, we see larger LoA. When considering raters' agreement (interrater reliability), higher bias is evident with a less impressive LoA between the measurements (FAST *R*^2^: −0.384, 0.291; FAST ICC: −0.388, 0.299).

## 4. Discussion

The aim of this study was to assess the test–retest and interrater reliability of the FAST outcomes. To mimic real-world practice in multicentre pharma-sponsored trials, we used inexperienced assessors to collect the data. Test–retest analysis revealed moderate to strong reliability. The interrater reliability results demonstrated weak to poor agreement between different assessors.

In the domain of pain psychophysics, QST allows researchers to characterize the somatosensory function in both healthy people and those experiencing pain.^3,5,26^ Quantitative sensory testing tasks can be categorized as 2 types: (1) Static measures, based on a response to a single stimulus (eg, thresholds, tolerance, or responses to a single suprathreshold stimulus). (2) Dynamic measures, characterized by the application of multiple stimuli (eg, temporal summation and conditioned pain modulation [CPM]).^3^ Although static measures have been showing moderate to good test–retest reliability,^8,13,14,20,27,29–31,33,35^ the studies assessing the reliability of dynamic QST measures have generally shown lower reliability. Few studies have found evidence of moderate to good test–retest reliability of temporal summation^8,13,14,20,27,29^ and CPM,^8,14,24^ whereas others have shown very low to moderate test–retest reliability across different dynamic QST tasks.^8,13,20,27,29,32,33,35^ Consequently, a systematic review of 10 different studies demonstrated that CPM reliability ranged from negligible to very strong.^24^ When focusing on interrater reliability, given the added source of variability arising from the between raters variability, the studies showed general lower reliability scores than for test–retest, observed for static and dynamic measures.^8,13,20,29–31^ Nonetheless, both static and dynamic measures have been widely adopted in clinical research.^36,37,43,45^

Our results are aligned with the moderate test–retest reliability findings of other dynamic measures, but the interrater indicators of reliability were in the low range of reliability. This could be partly explained by the differences in pain sensitivity observed between the 2 sessions. Differences in the raw pain intensity reports as captured during the FAST procedure between the 2 sessions could affect the WSV outcomes and, hence, their reliability.

This study of the reliability of the FAST was designed to mimic the real-world, pharma-sponsored, clinical trials for drug development. As commonly done in pharma-sponsored trials, we employed assessors without prior experience in QST measurement and had them undergo a short training session. Our interrater reliability findings emphasize the need to properly train staff members, especially in multisite studies, to improve the quality of data collection.

Other than its use as a screening tool in pharma-sponsored clinical trials, the FAST has also gained recent attention in theoretical research. Further substantiating our hypothesis presented in Kuperman et al.,^25^ a couple of studies have provided additional evidence supporting the idea that the WSV of pain reports may serve as a surrogate measure of the certainty of the ascending sensory information. One study demonstrated that dancers have lower WSV of their pain reports (as measured by the FAST). Their lower WSV was associated with years of practice and interoceptive sensibility, reflecting higher confidence in interpreting bodily sensations.^11^ Another study found associations between the FAST and the rubber hand illusion, a classical perceptual task that has recently been proposed to assess the same Bayesian brain hypothesis construct—the certainty of the ascending sensory information.^38^ Other computationally driven methods, such as those proposed by Drusko et al.,^15^ Hoskin et al.,^23^ and Wiech et al.,^44^ rely on extensive stimulus application (typically hundreds of noxious stimuli) and complex analyses, which reduce their adoption, compared with the FAST, which is easier to execute.

The FAST *R*^2^ outcome has been found to predict placebo response in patients with knee osteoarthritis and painful diabetic neuropathy in 2 clinical trials.^41,42^ Given these findings, the FAST has been used in pharma-sponsored trials as a screening tool to exclude patients with large WSV before enrolment in the trial. The 2 randomized controlled trials assessing the efficacy assessment of TRPV1 receptor antagonists in patients with osteoarthritis showed positive results after excluding patients with large WSV.^4,28^

Using a different approach, 2 other randomized controlled trials recruiting patients with painful diabetic neuropathy implemented an experimental pain-reporting training approach to decrease WSV of pain reports, as measured by the FAST.^2,42^ The first trial, evaluating pregabalin efficacy, demonstrated a reduction in the WSV of pain reports as a result of training, which led to a reduction in placebo response. Conversely, the second trial, which tested the combination of a novel histamine AZD5213 H3 receptor inverse agonist with pregabalin, showed a negative outcome, despite the implementation of similar training. A negative trial using the FAST does not mean that the test did not positively contribute. It might be that the drug under investigation was not effective. Unless the study is specifically designed to assess the added value of the FAST (this can be done only by comparing the results of 2 identical trials—one using the FAST as a screening tool and the other not using the FAST), we cannot reach unequivocal conclusions about the additive power of the FAST as a screening tool.

Since the development of the FAST tool,^40^ our understanding of the construct underlying this test and the terminology describing it have evolved. In the first years, we regarded the FAST as an assessment of pain reporting accuracy. We used this term, *pain reporting accuracy* because we assumed that most of the WSV observed in the FAST was due to error variability. However, the term *pain reporting accuracy* was, by definition, incorrect because accuracy is defined as “the closeness of a measure to its true value.” Given that we do not have a “true” pain value, we cannot ascribe *accuracy* to people's pain reports. In the past few years, we have consequently changed our terminology and now refer to the FAST outcomes as the *within-subject variability* of pain intensity reports. Although each of the 3 FAST outcomes represents a slightly different aspect of the variability observed in each subjects' FAST results, we do not yet have a clear understanding of these differences. Given that the *R*^2^ outcomes have demonstrated the strongest association with the placebo response in a few studies,^41,42^ we refer to *R*^2^ as the main FAST outcome (at least with regard to placebo research).

### 4.1. Limitations and future studies

One limitation of this study was that the time between sessions varied with the participants' availability. For this reason, we used time as a covariate in partial correlation, but this could not be controlled for in the calculation of the ICC. In addition, decrease in pain sensitivity from session 1 to session 2, which were observed in pain intensity reports in response to couple of the stimuli intensities, could reduce the reliability of the FAST outcomes. Another limitation was the unexperienced assessors, which may have contributed to the lower reliability of the main outcomes.

## 5. Conclusions

The FAST is an innovative experimental procedure for studying the WSV of pain intensity reports, and it has been implemented in both pharma-sponsored, multicentre, clinical trials and theoretical-based research. With inexperienced assessors, the FAST reliability is equivalent to that of other dynamic QST methods, with moderate to strong test–retest and weak-to-poor interrater reliability. For multisite applications, special attention should be devoted to the training of study staff. In theoretical-based, single-site studies conducted by experienced assessors, we expect the FAST to demonstrate better reliability.

## Disclosures

The authors have no conflict of interest to declare.
